# Estatinas e COVID-19: Suspender ou não Suspender? Eis a Questão!

**DOI:** 10.36660/abc.20200949

**Published:** 2021-01-27

**Authors:** Filipe Ferrari, Raul D. Santos

**Affiliations:** 1 Programa de Pós-Graduação em Cardiologia e Ciências Cardiovasculares Universidade Federal do Rio Grande do Sul Hospital de Clínicas de Porto Alegre Porto AlegreRS Brasil Programa de Pós-Graduação em Cardiologia e Ciências Cardiovasculares, Universidade Federal do Rio Grande do Sul, Hospital de Clínicas de Porto Alegre, Porto Alegre, RS - Brasil; 2 Instituto do Coração Faculdade de Medicina Universidade de São Paulo São PauloSP Brasil Unidade Clínica de Lípides, Instituto do Coração (InCor), Hospital da Faculdade de Medicina da Universidade de São Paulo, São Paulo, SP - Brasil; 3 Hospital Israelita Albert Einstein São PauloSP Brasil Hospital Israelita Albert Einstein, São Paulo, SP – Brasil

**Keywords:** COVID-19, Coronavírus, Betacoronavírus, Pandemia, Colesterol, Dislipidemias, Infecção, Inibidores de Hidroximetilglutaril-CoA Redutases, Lipoproteínas

## Introdução

Em meio a tantas incertezas que permeiam a nova doença de coronavírus 2019 (COVID-19), as evidências relacionando a presença de dislipidemia à gravidade da doença e às consequentes implicações prognósticas ainda são escassas. Em maio de 2020, um estudo chinês retrospectivo investigou a associação entre as mudanças nos níveis de colesterol e o prognóstico em aproximadamente 600 pacientes com COVID-19, pareados por idade e sexo com controles saudáveis. Primeiramente, observou-se que os níveis de colesterol de lipoproteína de baixa densidade (LDL-C) e colesterol total foram significativamente mais baixos em pacientes com COVID-19. Em segundo lugar, houve uma tendência para os níveis de LDL-C e de colesterol total diminuirem à medida que a gravidade da infecção aumentava (leve, grave e crítica, respectivamente).^1^ No mesmo estudo, os níveis de colesterol de lipoproteína de alta densidade (HDL-C) também diminuíram em casos graves. Foram observados dados parecidos por Fan et al.,^2^onde os níveis de LDL-C foram inversamente associados à gravidade da COVID-19. Esses dados sugeriram uma possível relação entre níveis baixos de colesterol e piora da infecção por COVID-19. Além disso, estudos experimentais têm mostrado que as estatinas podem aumentar a abundância da enzima conversora de angiotensina 2 (ECA2), o que pode contribuir parcialmente para a entrada do vírus na célula e o aumento do risco de infectividade.^3^

Com base nesses achados prévios, foi hipotetizado que o uso de terapias hipolipemiantes como as estatinas poderiam agravar a infecção por COVID-19. No entanto, sabe-se que os níveis de colesterol sérico podem cair em pacientes com infecções virais ou bacterianas ativas,^4 , 5^ uma vez que o LDL e o HDL desempenham um papel no sistema imunológico.^6^ Por outro lado, a hiperlipidemia pode comprometer a resposta imunológica e agravar ainda mais o estado inflamatório dos pacientes com COVID-19, aumentando o risco cardiovascular.^7^Desta maneira, surge a seguinte pergunta: as estatinas deveriam ou não ser suspensas em pacientes com COVID-19?

### COVID-19, Infecções, Trombose e Estatinas

#### Evidências de Benefícios Potenciais

Além de diminuir as lipoproteínas pró-aterogênicas, as estatinas apresentam outros efeitos sistêmicos bem documentados, como a melhora da disfunção endotelial e as propriedades anti-inflamatórias e anti-trombóticas que levam à estabilização das placas ateroscleróticas.^8^Meta-análises de ensaios clínicos randomizados mostraram que as estatinas podem reduzir significativamente as concentrações de proteína C reativa,^9^ antígeno do fator de von Willebrand^10^ e endotelina-1.^11^

Um estudo observacional com 3.043 pacientes hospitalizados pelo vírus influenza encontrou menor risco de mortalidade naqueles que estavam em uso de estatinas, antes ou durante a hospitalização (odds ratio [OR] ajustado 0,59).^12^Também foi observado um benefício das estatinas em pacientes hospitalizados com pneumonia viral, resultando em menor mortalidade e necessidade de intubação (OR 0,26).^13^

Dado o estado pró-inflamatório e pró-trombótico observado em pacientes com COVID-19 mais grave, as características dessas drogas podem ser importantes para esses pacientes.

A Tabela 1 mostra detalhes de alguns estudos que examinaram os efeitos das estatinas em pacientes com infecções virais e COVID-19.

**Tabela 1:** – Evidências de possíveis benefícios das estatinas no cenário de doenças virais, bem como na COVID-19

Estudo	Desenho do Estudo	Pacientes e Doença	Total (N) Idade média	Ajuste para covariáveis	Resultados
Vandermeer et al. 2011 ^12^	Multiestado	Pacientes hospitalizados com infecções pelo vírus influenza	3.043 70 anos	Idade, raça, DCV, doença pulmonar e renal, vacinação contra influenza e administração de medicamentos antivirais	Estatinas antes ou durante a hospitalização versus nenhuma estatina foram associadas a chances de proteção contra a mortalidade em 30 dias OR ajustado 0,59; IC 95%, 0,30 a 0,92
Henry et al. 2018 ^13^	Retrospectivo	Pacientes com pneumonia viral	539 64 anos	NA	Estatinas continuadas no hospital versus descontinuação reduziram mortes e/ou necessidade de intubação durante a internação hospitalar OR 0,26; IC 95%, 0,08 a 0,81; p = 0,02
De Spiegeleer et al. 2020 ^14^	Coorte multicêntrica retrospectiva	Sujeitos positivos para COVID-19	154 86 anos	Idade, sexo, estado funcional, hipertensão e diabetes mellitus	O uso de estatinas foi relacionado à ausência de sintomas durante COVID-19 OR 2,91; IC 95%, 1,27 a 6,71; p = 0,011 OR ajustado 2,65; IC 95%, 1,13 a 6,68; p = 0,028
Zhang et al. 2020 ^15^	Retrospectivo	Pacientes hospitalizados por COVID-19	13.981 58 anos	Idade, sexo e SpO2 no momento da admissão	Uso de estatinas versus nenhuma estatina foi correlacionado à redução no risco de mortalidade por todas as causas em 28 dias HR ajustado 0,58; IC 95%, 0,43 a 0,80; p = 0,001
Rodriguez-Nava et al. 2020 ^16^	Coorte retrospectiva	Pacientes com COVID-19 admitidos à unidade de terapia intensiva	87 68 anos	Idade, hipertensão, DCV, ventilação mecânica invasiva, frequência respiratória > 30, SpO2 < 94%, PaO2 / FiO2 < 300 mmHg ou infiltrados pulmonares > 50%, número de comorbidades e outras terapias adjuvantes (incluindo hidroxicloroquina, esteróides intravenosos, azitromicina, tocilizumabe, colchicina e antibióticos)	O uso de estatina (especificamente atorvastatina) reduziu a progressão à morte HR ajustado 0,38, IC 95%, 0,18 a 0,77; p = 0,008
Daniels et al. 2020 ^17^	Unicêntrico retrospectivo	Pacientes hospitalizados por COVID-19	170 59 anos	Idade, sexo, obesidade, hipertensão, diabetes, doença renal crônica e DCV	Uso de estatinas antes da admissão reduziu o desenvolvimento de doença grave OR ajustado 0,29; IC 95%, 0,11 a 0,71; p = 0,009 O uso de estatinas aumentou a taxa de recuperação da COVID-19 entre os indivíduos que ainda não haviam experimentado doença grave HR ajustado por causa específica para recuperação 2,69; IC 95%, 1,36 a 5,33; p = 0,004
Song et al. 2020 ^18^	Coorte retrospectiva	Pacientes hospitalizados por COVID-19	249 62 anos	Idade, sexo, raça, DCV, doença pulmonar crônica, diabetes e obesidade	O uso de estatinas diminuiu o risco de ventilação mecânica invasiva OR ajustado 0,45; IC 95%, 0,20 a 0,99; p = 0,048

*OR: odds ratio; HR: hazard ratio; IC: intervalo de confiança; SpO_*2*_: saturação periférica de oxigênio: DCV: doença cardiovascular; NA: não aplicável.*

Em um estudo de coorte retrospectivo da Bélgica, De Spiegeleer et al.^14^ avaliaram 154 pessoas idosas (idade média: 86 anos) que contraíram COVID-19, observando uma tendência significativa para ausência de sintomas naqueles que recebiam estatinas anteriormente (OR 2,91; intervalo de confiança (IC) 95%, 1,27 a 6,71). Isto permaneceu estatisticamente significativo mesmo após o ajuste para covariáveis (OR 2,65; IC 95%, 1,13 a 6,68).

Outro estudo retrospectivo de aproximadamente 14.000 pacientes com COVID-19 encontrou menor risco de mortalidade com o uso prévio de estatinas. Neste estudo, 1.219 pacientes estavam recebendo estatinas e a mortalidade geral em 28 dias neste grupo foi de 5,2%, enquanto no grupo sem estatinas foi de 9,4% (hazard ratio [HR] ajustado 0,58; IC 95%, 0,43 a 0,80; p = 0,001).^15^ Em outro estudo com 87 pacientes com COVID-19 internados na unidade de terapia intensiva, uma progressão mais lenta para a morte foi encontrada naqueles que receberam atorvastatina.^16^

Daniels et al.,^17^ por meio de um estudo retrospectivo de centro único, encontraram um risco reduzido de COVID-19 grave em pacientes que estavam recebendo estatinas antes da admissão (OR ajustado 0,29) e um tempo mais rápido de recuperação entre aqueles sem doença grave (HR ajustado para recuperação 2,69). Além disso, em um estudo de coorte retrospectivo de pacientes hospitalizados com COVID-19 (N = 249) nos Estados Unidos, o uso de estatinas foi correlacionado à diminuição do risco de ventilação mecânica invasiva (OR ajustado 0,45).^18^

Certamente, os estudos citados são severamente limitados por seu desenho retrospectivo; esses dados, apesar de favoráveis ao uso de estatinas em infecções virais, são apenas geradores de hipóteses, podendo estar sujeitos a um viés de seleção de indivíduos que recebem melhores cuidados. A questão que se segue é: há alguma evidência de que as estatinas possam prevenir doenças infecciosas? Em uma análise post hoc de pacientes incluídos no ensaio JUPITER,^19^que randomizou 17.802 indivíduos com LDL-C < 130 mg/dL e proteína C reativa de alta sensibilidade ≥ 2,0 mg/L para receber rosuvastatina 20 mg/dia ou placebo, seguidos por tempo mediano de 1,9 anos, Novack et al.^20^ observaram que o uso de estatinas reduziu, embora modestamente, a incidência de pneumonia (HR 0,83, IC 95%, 0,69 a 1,00). Esses resultados, que merecem ser comprovados em um ensaio adequadamente delineado, sugerem que as estatinas possam reduzir o risco de pneumonia devido a possíveis efeitos benéficos leves anti-inflamatórios, antioxidantes, imunomoduladores, anti-apoptóticos e endoteliais, segundo os autores.^18^É incerto se isso beneficiaria pacientes com COVID-19.

**Figura 1:**
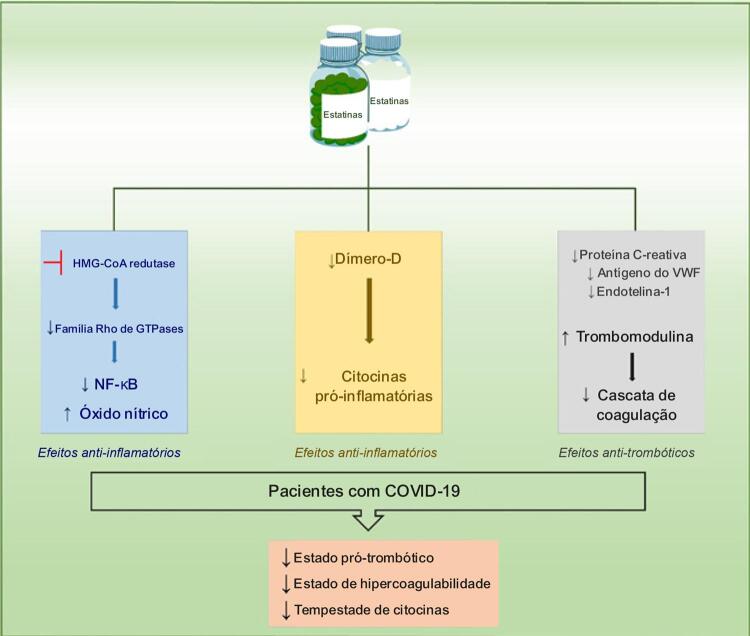
– Alguns mecanismos propostos por meio dos quais as estatinas reduzem o estado pró-inflamatório e pro-trombótico em pacientes com COVID-19. ^8^ - ^11^ , ^24^ , ^25^ HMG-CoA redutase: 3-hidroxi-3-methyl-glutaril-CoA redutase; NF-κB: fator nuclear kappa B; VWF: fator de von Willebrand.

Além das complicações pulmonares, o SARS-CoV-2 também pode induzir a trombose.^21^ As estatinas teriam efeitos benéficos nestes casos? Em uma análise pré-especificada do mesmo ensaio JUPITER,^19^ foi analisado o impacto da rosuvastatina na primeira ocorrência de embolia pulmonar ou de tromboembolismo venoso. Embora não tenha havido diferenças nas taxas de embolia pulmonar entre os grupos (rosuvastatina e placebo), o grupo que recebeu a estatina apresentou uma redução de 43% nas taxas de tromboembolismo venoso (HR 0,57; IC 95%, 0,37 a 0,86; p = 0,007).^22^ Além disso, uma metanálise de 13 estudos de coorte observacionais (N = 3.148.259) e 23 ensaios clínicos randomizados (N = 118.464) mostrou que, tanto em estudos de coorte observacionais quanto em ensaios clínicos randomizados, houve uma redução no risco de trombose venosa profunda, mas não de embolia pulmonar, quando o uso de estatina foi comparado com os controles (risco relativo [RR] 0,75; IC 95%, 0,65 a 0,87; p < 0,0001; 0,85; IC 95%, 0,73 a 0,99; p = 0,038). Um benefício maior também foi encontrado para o risco de tromboembolismo venoso com o uso de rosuvastatina em comparação com outras estatinas (RR 0,57; IC 95%, 0,22 a 0,75; p = 0,015).^23^Possíveis mecanismos para explicar esses resultados incluem os efeitos das estatinas sobre fatores pró-trombóticos, como a redução do dímero D, fator VIII,^24^ inibidor do ativador do plasminogênio tipo 1 e níveis de fator tecidual, bem como a diminuição da agregação plaquetária e o aumento da expressão de trombomodulina.^25^A Figura 1 apresenta alguns mecanismos propostos onde as estatinas podem atuar como agentes anti-trombóticos e anti-inflamatórios e podem exercer efeitos favoráveis em pacientes com COVID-19.

Visto que uma porção não negligenciável de pacientes infectados pelo SARS-CoV-2 (especialmente os pacientes mais graves) pode apresentar alterações no sistema de coagulação e uma alta taxa de tromboembolismo venoso,^26^ a manutenção das estatinas pode melhorar o prognóstico desses indivíduos. No entanto, da mesma forma que as possíveis propriedades anti-infecciosas, isso também precisa ser confirmado em ensaios clínicos randomizados.

## Suspensão de Estatinas e Aumento do Risco de Eventos Cardiovasculares?

A preocupação de que os níveis baixos de colesterol possam ser deletérios para pacientes com COVID-19 pode levar à suspensão inadequada de medicamentos hipolipemiantes em pacientes com alto risco de doença cardiovascular. As estatinas são a base fundamental para a terapia hipolipemiante com o objetivo de reduzir o risco de doença arterial coronariana (DAC); como grupo, as estatinas constituem um dos medicamentos mais prescritos no mundo. A metanálise do Cholesterol Treatment Trialists (CTT)^27^ mostrou que para cada redução de 1,0 mmol/L (~ 40 mg/dL) de LDL-C, houve uma redução de 10% na mortalidade por todas as causas (RR 0,90, IC 95%, 0,87 a 0,93; p < 0,0001), além de uma redução de 20% nas mortes por DAC (RR 0,80; IC 99%, 0,74 a 0,87; p < 0,0001).

Um cenário importante em que a suspensão de estatinas pode ser deletéria é durante o período inicial após um evento de síndrome coronariana aguda. Nesse cenário, a adição e manutenção de estatinas são fundamentais, e a suspensão do medicamento pode aumentar os riscos dos pacientes. Nesse sentido, um estudo observacional brasileiro com 249 pacientes observou um efeito inflamatório rebote na fase aguda do infarto do miocárdio (IM) após a retirada das estatinas. Sposito et al.^28^verificaram que, no começo do estudo, os pacientes que recebiam estatinas apresentavam valores de proteína C-reativa mais baixos quando comparados aos que não recebiam, antes do início do IM. No quinto dia após o IM, a mediana da proteína C reativa foi significativamente mais alta no grupo onde as estatinas foram suspensas.^28^ Em adição a isso, em uma análise de pacientes que apresentaram com DAC e dor no peito durante as últimas 24 horas no estudo PRISM^29^ (N = 1.616), Heeschen et al.^30^relataram que o uso de estatinas reduziu a taxa de eventos após 30 dias, em comparação com os pacientes sem esses medicamentos (HR ajustado 0,49, IC 95%, 0,21 a 0,86). Quando as estatinas foram suspensas após a admissão, o risco cardíaco aumentou (OR 2,93; IC 95%, 1,64 a 6,27; p = 0,005) e, embora não tenha sido estatisticamente significativo, houve uma tendência de maior risco em comparação com os pacientes que nunca receberam estatinas (OR 1,69; IC 95%, 0,92 a 3,56).^29^ Portanto, a retirada desses medicamentos deve ser vista com extrema cautela, principalmente após um evento coronariano agudo, pois pode levar ao surgimento de complicações, piorando o prognóstico dos pacientes.

Em suma, o uso de estatinas é baseado em uma literatura sólida e robusta, e a sua suspensão, exceto por indicação médica, pode levar a eventos agudos, aumentando ainda mais os riscos dos pacientes infectados por COVID-19, especialmente aqueles em prevenção secundária e aqueles que tiveram um evento coronário agudo recente. Os médicos e os pacientes devem manter esse conhecimento em mente.

## Quando Devemos Considerar a Suspensão das Estatinas em Pacientes com COVID-19?

De acordo com as diretrizes da Sociedade Europeia de Cardiologia, em casos raros em que os pacientes com COVID-19 desenvolvem rabdomiólise grave ou aumento das enzimas hepáticas, a suspensão temporária da terapia com estatinas é prudente.^31 , 32^ Além disso, se o paciente estiver em risco iminente de vida, a suspensão deve ser realizada, pelo menos até a recuperação da infecção.^33^

## Conclusões

O uso de estatinas é apoiado por uma literatura sólida, com inquestionáveis benefícios cardiovasculares. Apesar das evidências de que concentrações mais baixas de colesterol estão associadas a um curso mais grave de COVID-19, não há, entretanto, evidências de que as estatinas possam piorar o prognóstico. Ao contrário, essas drogas podem reduzir os mecanismos pró-inflamatórios e pró-trombóticos que caracterizam os casos mais graves de COVID-19. Atualmente, não existem evidências apoiando a descontinuação das estatinas em pacientes com COVID-19, exceto quando ocorrem elevações importantes das enzimas hepáticas, rabdomiólise ou risco de vida atribuído ao medicamento. Por outro lado, não há indicação para o uso desses medicamentos especificamente para prevenir complicações da infecção pelo SARS-CoV-2.
