# Supplementary material for: Estatinas e COVID-19: Suspender ou não Suspender? Eis a Questão!
Source: Arq Bras Cardiol. 2021 Jan 27;116(1):147–52. [Article in Portuguese] doi: 10.36660/abc.20200949 (PMC8159509; doi:10.36660/abc.20200949)
Supplement: Translation

# Translation: Statins and COVID-19: To Suspend or Not to Suspend? That is the Question!

**Authors:** Filipe Ferrari, Raul D. Santos

**Affiliations:** 1 Brazil Graduate Program in Cardiology and Cardiovascular Sciences, Universidade Federal do Rio Grande do Sul, Hospital de Clínicas de Porto Alegre, Porto Alegre, RS – Brazil; 2 Brazil Lipid Clinic Heart Institute (InCor), University of São Paulo Medical School Hospital, São Paulo, SP – Brazil; 3 Brazil Hospital Israelita Albert Einstein, São Paulo, SP – Brazil

**Keywords:** COVID-19, Coronavirus, Betacoronavirus, Pandemics, Cholesterol, Dyslipidemias, Infection, Dydroxymethylglutaryl-CoA Reductase Inhibitors, Lipoproteins

## Introduction

In the midst of so many uncertainties that permeate the new coronavirus disease 2019 (COVID-19), the evidence relating the presence of dyslipidemia to disease severity and consequent prognostic implications are still scarce. In May 2020, a retrospective Chinese study investigated the association between changes in cholesterol levels and prognosis in approximately 600 patients with COVID-19, who were paired by age and sex with healthy controls. First, it was observed that low-density lipoprotein cholesterol (LDL-C) and total cholesterol levels were significantly lower in patients with COVID-19. Second, there was a trend for LDL-C and total cholesterol levels to decrease as the severity of infection increased (mild, severe, and critical, respectively).^1^In that study, high-density lipoprotein cholesterol (HDL-C) levels were also decreased in severe cases. Similar data were observed by Fan et al.,^2^where levels of LDL-C were inversely associated with the severity of COVID-19. These data suggested a possible relation between low cholesterol levels and worsening of COVID-19 infection. In addition, experimental studies have shown that statins might increase the abundance of the angiotensin-converting enzyme 2 (ACE2), which could in part contribute to the entry of the virus into the cell and increase the risk of infectivity.^3^

Based on these previous findings, it was hypothesized that use of lipid-lowering therapies like statins could aggravate COVID-19 infection. However, it is known that serum cholesterol levels may drop in patients with active viral or bacterial infections,^4 , 5^since LDL and HDL have a role in the immune system.^6^On the other hand, hyperlipidemia can compromise the immune response and further exacerbate the inflammatory status of COVID-19 patients, increasing cardiovascular risk.^7^So, the question that ensues is, should statins be suspended or not in patients with COVID-19?

### COVID-19, Infections, Thrombosis, and Statins

#### Evidence of Potential Benefit

In addition to lowering pro-atherogenic lipoproteins, statins have other well-documented systemic effects, such as improvement in endothelial dysfunction, as well as anti-inflammatory and anti-thrombotic properties that lead to stabilization of atherosclerotic plaques.^8^Meta-analyses of randomized clinical trials have shown that statins can significantly reduce concentrations of C-reactive protein,^9^von Willebrand factor antigen,^10^and endothelin-1.^11^

An observational study with 3,043 patients hospitalized for the influenza virus found a lower risk of mortality in those using statins, before or during hospitalization (adjusted odds ratio [OR] 0.59).^12^Benefit from statins was also observed in hospitalized patients with viral pneumonia, resulting in lower mortality and need for intubation (OR 0.26).^13^

Given the pro-inflammatory and pro-thrombotic status observed in patients with more severe COVID-19, the characteristics of these drugs may be important for these patients.

Table 1 shows details of some studies examining the effects of statins in patients with viral infections and COVID-19.

**Table 1:** – Evidence of Possible Benefits of Statins in the Viral Disease Scenario, as well as in COVID-19

Study	Study Design	Patients and Disease	Total (N) Mean Age	Adjustment for Covariates	Results
Vandermeer et al. 2011 ^12^	Multistate	Patients hospitalized with influenza virus infections	3,043 70 years	Age, race, CVD, lung and renal disease, influenza vaccination, and antiviral administration	Statins prior or during hospitalization versus no statin were associated with a protective odds of death within 30 days Adjusted OR 0.59; 95% CI, 0.30 to 0.92
Henry et al. 2018 ^13^	Retrospective	Patients with viral pneumonia	539 64 years	NA	Statins continued in hospital versus discontinuation reduced death and/or need of intubation throughout the hospital stay OR 0.26; 95% CI, 0.08 to 0.81; P = 0.02
De Spiegeleer et al. 2020 ^14^	Retrospective multicenter cohort	COVID-19–positive subjects	154 86 years	Age, sex, functional status, hypertension, and diabetes mellitus	The use of statins was related to the absence of symptoms during COVID-19 OR 2.91; 95% CI, 1.27 to 6.71; P = 0.011 Adjusted OR 2.65; 95% CI, 1.13 to 6.68; P = 0.028
Zhang et al. 2020 ^15^	Retrospective	Patients hospitalized for COVID-19	13,981 58 years	Age, sex, and SpO2 at admission	Use of statins versus no statin was correlated to the reduction in the risk for 28-day all-cause mortality Adjusted HR 0.58; 95% CI, 0.43 to 0.80; P = 0.001
Rodriguez-Nava et al. 2020 ^16^	Retrospective cohort	Patients with COVID-19 admitted to intensive care unit	87 68 years	Age, hypertension, CVD, invasive mechanical ventilation, respiratory rate > 30, SpO2 < 94%, PaO2/FiO2 < 300 mmHg or lung infiltrates > 50%, number of comorbidities, and other adjuvant therapies (including hydroxychloroquine, intravenous steroids, azithromycin, tocilizumab, colchicine, and antibiotics)	The use of statin (specifically atorvastatin) has reduced the progression to death Adjusted HR 0.38, 95% CI, 0.18 to 0.77; P = 0.008
Daniels et al. 2020 ^17^	Retrospective single-center	Patients hospitalized for COVID-19	170 59 years	Age, sex, obesity, hypertension, diabetes, chronic kidney disease and CVD	Use of statins prior to admission reduced development of severe disease Adjusted OR 0.29; 95% CI, 0.11 to 0.71; p = 0.009 Statin use increased rate of recovery from COVID-19 among subjects who had not yet experienced severe disease Cause-specific adjusted HR for recovery 2.69; 95% CI, 1.36 to 5.33; p = 0.004
Song et al. 2020 ^18^	Retrospective cohort	Patients hospitalized for COVID-19	249 62 years	Age, sex, race, CVD, chronic pulmonary disease, diabetes, and obesity	Statin use decreased risk for invasive mechanical ventilation Adjusted OR 0.45; 95% CI, 0.20 to 0.99; p = 0.048

*OR: odds ratio; HR: hazard ratio; CI: confidence interval; SpO_*2*_: peripheral oxygen saturation; CVD: cardiovascular disease; NA: not applicable.*

In a retrospective cohort study from Belgium, De Spiegeleer et al.^14^evaluated 154 elderly people (mean age: 86 years) who contracted COVID-19, and observed a significant trend for absence of symptoms in those previously taking statins (OR 2.91; 95% confidence interval (CI), 1.27 to 6.71). This remained statistically significant even after adjusting for covariates (OR 2.65; 95% CI, 1.13 to 6.68).

Another retrospective study of approximately 14,000 patients with COVID-19 found a lower risk of mortality with previous use of statins. In this study, 1,219 patients were receiving statins, and the all-cause mortality at 28 days in this group was 5.2%, while in the non-statin group it was 9.4% (adjusted hazard ratio [HR] 0.58; 95% CI, 0.43 to 0.80; p = 0.001).^15^In another study with 87 patients with COVID-19 admitted to the intensive care unit, a slower progression to death was found in those receiving atorvastatin.^16^

Daniels et al.,^17^through a retrospective single-center study, found a reduced risk of severe COVID-19 in patients who were using statins prior to admission (adjusted OR 0.29), and a faster time to recovery among those without severe disease (HR adjusted for recovery 2.69). In addition, in a retrospective cohort study of patients hospitalized with COVID-19 (N = 249) in the United States, the use of statins correlated with decreased risk for invasive mechanical ventilation (adjusted OR 0.45).^18^

Of course, the quoted studies are severely limited by their retrospective design; these data, despite being favorable to use of statins in viral infections, are only hypothesis generating, and they may be subject to a selection bias of individuals receiving better care. The question that ensues is, would there be any evidence that statins may prevent infectious diseases? In a post hoc analysis of patients included in the JUPITER trial,^19^which randomized 17,802 individuals with LDL-C < 130 mg/dL and high-sensitivity C-reactive protein ≥2.0 mg/L to receive rosuvastatin 20 mg/day or placebo followed for a median of 1.9 years, Novack et al.^20^observed that the use of statins reduced, albeit modestly, the incidence of pneumonia (HR 0.83, 95% CI, 0.69 to 1.00). These results, which deserve to be proven in an adequately designed trial, suggest that statins may reduce pneumonia risk due to possible beneficial mild anti-inflammatory, antioxidant, immunomodulatory, anti-apoptotic, and endothelial effects according to the authors.^18^Whether this would benefit patients with COVID-19 is uncertain.

In addition to pulmonary complications, SARS-CoV-2 may also induce thrombosis.^21^Would statins have beneficial effects in these cases? In a pre-specified analysis of the same JUPITER trial,^19^the impact of rosuvastatin on the first occurrence of pulmonary embolism or venous thromboembolism was analyzed. Although there were no differences in the rates of pulmonary embolism between the groups (rosuvastatin and placebo), the group that received the statin showed a 43% reduction in the rates of venous thromboembolism (HR 0.57; 95% CI, 0.37 to 0.86; p = 0.007).^22^Furthermore, a study-level meta-analysis of 13 observational cohort studies (N = 3,148,259) and 23 randomized clinical trials (N = 118,464) showed that, in both observational cohort studies and randomized clinical trials, there was a reduction in risk of deep venous thromboembolism but not of pulmonary embolism, when statin use was compared with controls (relative risk [RR] 0.75; 95% CI, 0.65 to 0.87; p < 0.0001; 0.85; 95% CI, 0.73 to 0.99; p = 0.038). A greater benefit was also found for the risk of venous thromboembolism with the use of rosuvastatin compared to other statins (RR 0.57; 95% CI, 0.22 to 0.75; p = 0.015).^23^Possible mechanisms to explain these results include the effects of statins on pro-thrombotic factors, such as reduced D-dimer, factor VIII,^24^plasminogen activator inhibitor 1, and tissue factor levels, as well as decreased platelet aggregation and increased expression of thrombomodulin.^25^ Figure 1 presents some proposed mechanisms where statins may act as antithrombotic and anti-inflammatory agents and could exert favorable effects in patients with COVID-19.

**Figure 1:** – Some proposed mechanisms for statins to reduce pro-inflammatory and prothrombotic state in patients with COVID-19. ^8^ - ^11^ , ^24^ , ^25^ HMG-CoA reductase: 3-hydroxy-3-methylglutaryl-CoA reductase; NF-κB: nuclear factor kappa B; VWF: von Willebrand factor.

Since a non-negligible portion of patients infected by SARS-CoV-2 (especially the more severe patients) may present alterations in the coagulation system and a high rate of venous thromboembolism,^26^the maintenance of statins may improve these individuals’ prognosis. However, similarly to the possible anti-infectious properties, this also needs to be confirmed in randomized clinical trials.

## Statin Suspension and Increased Risk of Cardiovascular Events?

The concern that low cholesterol levels could be deleterious to patients with COVID-19 may lead to inappropriate suspension of lipid lowering medications in patients at high risk of cardiovascular disease. Statins are the cornerstone for lipid lowering therapy with the aim of reducing the risk of coronary artery disease (CAD); as a group, statins are one of the most prescribed drugs in the world. The Cholesterol Treatment Trialists Meta-analysis (CTT)^27^showed that for each 1.0 mmol/L (~ 40 mg/dL) reduction of LDL-C, all-cause mortality was reduced by 10% (RR 0.90, 95% CI, 0.87 to 0.93; p < 0.0001), in addition to a 20% reduction in CAD deaths (RR 0.80; 99% CI, 0.74 to 0.87; p < 0.0001).

An important scenario where statin suspension could be deleterious is during the early period after an acute coronary syndrome event. In this scenario, the addition and maintenance of statins are fundamental, and drug suspension may increase patients’ risks. In this sense, a Brazilian observational study with 249 patients observed a rebound inflammatory effect in the acute phase of myocardial infarction (MI) after statin withdrawal. Sposito et al.^28^found that, at the beginning of the study, those who were receiving statins had lower C-reactive protein values when compared to those who were not, before the onset of MI. On the fifth day after MI, median C-reactive protein was significantly higher in the group where statins had been suspended.^28^In addition, in an analysis of patients presenting with CAD and chest pain within the last 24 hours in the PRISM study^29^(N = 1,616), Heeschen et al.^30^reported that the use of statins reduced the rate of events after 30 days, compared to patients without those medications (adjusted HR 0.49, 95% CI, 0.21 to 0.86). When statins were suspended after admission, cardiac risk increased (OR 2.93; 95% CI, 1.64 to 6.27; p = 0,005), and, although it was not statistically significant, there was a trend to greater risk compared to patients who had never received statins (OR 1.69; 95% CI, 0.92 to 3.56).^29^Therefore, the withdrawal of these drugs should be viewed with extreme caution, especially after an acute coronary event, since this may lead to appearance of complications, worsening patients’ prognosis.

In short, the use of statins is based on solid and robust literature, and their discontinuation, except for medical indication, may lead to acute events, further increasing the risk of patients infected by COVID-19, especially of those in secondary prevention and those who have had a recent acute coronary event. Physicians and patients should keep this knowledge in mind.

## When Should We Consider Suspending the Statins in Patients with COVID-19?

According to European Society of Cardiology guidelines, in rare cases where patients with COVID-19 develop severe rhabdomyolysis or increased liver enzymes, temporary suspension of statin therapy is prudent.^31 , 32^Furthermore, if the patient is at imminent risk of life, suspension should be carried out, at least until recovery from the infection.^33^

## Conclusions

The use of statins is supported by solid literature, with unquestionable cardiovascular benefits. Despite evidence that lower cholesterol concentrations are associated with more severe course of COVID-19, there is, however, no evidence that statins may worsen prognosis. On the contrary, these drugs may reduce the pro-inflammatory and pro-thrombotic mechanisms that characterize more severe cases of COVID-19. Currently, there is no evidence to support discontinuation of statins in patients with COVID-19, except when important elevations of hepatic enzymes, rhabdomyolysis, or drug-attributed risk of life occur. On the other hand, there is no indication for the use of these drugs specifically to prevent complications of SARS-CoV-2 infection.
